# Development and Preliminary Verification of a Mandarin-Based Hearing-Aid Fitting Strategy

**DOI:** 10.1371/journal.pone.0080831

**Published:** 2013-11-20

**Authors:** Ying-Hui Lai, Tien-Chen Liu, Pei-Chun Li, Wan-Ting Shih, Shuenn-Tsong Young

**Affiliations:** 1 Research Center for Information Technology Innovation, Academia Sinica, Taipei, Taiwan; 2 Holistic Education Center, Mackay Medical College, New Taipei City, Taiwan; 3 Department of Audiology and Speech Language Pathology, Mackay Medical College, New Taipei City, Taiwan; 4 Department of Otolaryngology, National Taiwan University Hospital, Taipei, Taiwan; McGill University, Canada

## Abstract

**Objective:**

The purpose of this study was to design and to verify a new hearing-aid fitting strategy (Aescu HRL-1) based on the acoustic features of Mandarin. The subjective and objective outcomes were compared to those fitted with NAL-NL1 (National Acoustic Laboratory Non-Linear, version1) in Mandarin-speaking hearing-aid users.

**Design:**

Fifteen subjects with sensorineural hearing loss participated in this preliminary study. Each subject wore a pair of four-channel hearing aids fitted with the Aescu HRL-1 and NAL-NL1 prescriptions alternatively for 1 month. Objective and subjective tests including the Mandarin Monosyllable Recognition Test (MMRT), Mandarin Hearing in Noise Test (MHINT), International Outcome Inventory for Hearing Aids (IOI-HA), and a sound-quality questionnaire were used to evaluate the performance of the two prescriptions.

**Results:**

The mean MMRT scores were 79.9% and 81.1% for NAL-NL1 and Aescu HRL-1 respectively. They are not statistically different. The corresponding MHINT signal-to-noise ratios were 0.87 and 0.85 dB, also, no significant difference was found between these two strategies. However, in subjective questionnaires, overall, the sound-quality and IOI-HA scores were higher for Aescu HRL-1.

**Conclusions:**

The speech recognition performance based on Aescu HRL-1 is as good as that of NAL-NL1 for Mandarin-speaking hearing-aid users. Moreover, the subjects generally responded that Aescu HRL-1 provides a more natural, richer, and better sound quality than does NAL-NL1.

## Introduction

Over the past 60 to 70 years, and especially during the most recent 35 years, there has been substantial research into the amplification needs of people with hearing impairments. Accordingly, many fitting strategies (so-called prescriptions) for hearing aids have been developed to ensure that incoming sounds are audible whilst not being excessively loud for the hearing-aid users [1-3]. So far, nonlinear amplification is the most commonly used prescription method. It involves compression of the dynamic range of incoming sounds into the residual auditory dynamic range of a hearing-impaired (HI) individual. Nonlinear amplification is also called wide-dynamic-range compression (WDRC), and it is widely accepted that compression amplification can be beneficial – at least in maintaining speech intelligibility and comfortable listening – over a range of input conditions [4]. Nonlinear amplification fitting strategies can be roughly divided into two categories based on their prescription rationales: loudness normalization and loudness equalization [5]. Loudness normalization was rapidly employed for fitting multichannel WDRC hearing aids, and it provides sufficient amplification to all frequencies so that the hearing-aid user perceives the full range of sounds at the same loudness as does a person with normal hearing (NH). This is based on the reasonable assumption that HI subjects will be satisfied with and will benefit from having the loudness perception of NH listeners [6,7]. Popular prescriptions based on loudness normalization include IHAFF (Independent Hearing Aid Fitting Forum) [8], and DSL (Desired Sensation Level) [9]. However, it is well known that the energy content of speech is greater at low frequencies, and that the low-frequency content of steady background noises is typically higher than that of speech. Keidser and Grant [6] argued that applying loudness normalization to hearing-aid fittings may result in both the speech and background noise being dominated by low-frequency energy, and the hearing-aid user may therefore suffer from the effect of upward spread of masking, which reduces the speech intelligibility, especially for low-level, high-frequency speech components.

This situation led to the other prescription (loudness equalization) being proposed in order to reduce the effect of upward spread of masking by presenting speech bands equally loud. Loudness equalization does not aim at normalizing frequency-specific loudness. Instead, the goal is to maximize speech intelligibility for every input level with the constraint that the overall loudness of speech must not exceed the normal level [6]. The most popular fitting strategy based on loudness equalization are such as: National Acoustic Laboratories – Nonlinear Fitting, version 1 (NAL-NL1) [10,11], this prescription applies a lower gain to low frequencies, even for someone with a flat hearing loss. NAL-NL1 aims to maximize speech intelligibility for any input level above the compression threshold while making the overall loudness of the speech equal to or less than normal. It was derived by calculations combining a loudness model [12] with a modified form of the Speech Intelligibility Index (SII) [13]. The SII used in the NAL-NL1 fitting strategy was calculated from the data of Ching et al.[14], and these data were based on Bamford-Kowal-Bench sentence lists [14,15]. Keidser and Grant’s studies have found that hearing-aid prescriptions based on NAL-NL1 can provide better speech intelligibility than those based on loudness normalization when listening in a low-frequency weighted background noise and makes speech clearer in many environments [5,6,16]; however, most hearing-aid users describe loudness normalization prescription (i.e., IHAFF) as “comfortable” and “natural”. Greater emphasis is placed on loudness and sound quality than on speech intelligibility when using loudness normalization [17]. Therefore, the sound quality and speech recognition could differ between hearing aids using these two prescriptions [7]. A few new fitting strategies were proposed in recent years, such as NAL-NL2 [11] and CAMEQ2-HF [18]. NAL-NL2 attempts to maximize speech intelligibility by using a new intelligibility model and new concepts of compression ratio setting. The CAMEQ2-HF attempts to recommend more gains for center frequencies. It also adopts a new diffuse-field-to-eardrum transfer function and new measurement methods to evaluate the average spectrum of speech. The performance and benefits of these new fitting strategies were gradually accumulating [18-21]. 

 Approximately 1.2 billion people currently use Mandarin to communicate [22], but no hearing-aid prescriptions are based specifically on the characteristics of Mandarin. The differences of acoustic characteristics between English and Mandarin are controversial. Several studies have found no systematic separation between English compared with non-English languages, or between tonal compared with non-tonal languages [23]. For example, McCullough et al. found no difference between the long-term average speech spectra of English and Mandarin [24]. In contrast, some other studies have demonstrated that Mandarin is different from English. Mandarin words are monosyllabic while English words can be monosyllabic, disyllabic, or multisyllabic. In addition, unlike English, Mandarin is a tonal language, where different tones distinguish different meanings [25]. Also, the frequency importance function, which is widely held that suggests certain parts of the auditory spectrum are more important than others for speech recognition, also differs between Mandarin and English. Chen [26] showed that frequencies from 2000 to 4000 Hz are more important in Mandarin speech than in English speech. These controversial results could be due to many possible reasons, such as the difference in talkers, speech materials, recording equipment and analysis procedures.

Our laboratory has focused on the acoustic characteristics of Mandarin in recent years. Our results indicate that the frequency importance function of Mandarin is more weighted than English in the frequency band of 1000 to 6300 Hz, which has the summed importance of 72% in this band [27]; in addition, the speech map of Mandarin is also different from English, especially at the frequency of 125 and 2000 Hz [28]. Therefore, we hypothesize that hearing-aid fitting strategy based on the characteristics of English might not be optimal for Mandarin speakers. A new strategy can be designed to incorporate the acoustic characteristic of Mandarin.

The main purpose of this study was to design a loudness normalization based fitting strategy according to the acoustic characteristic of Mandarin speech map. The strategy is called Aescu Hearing Research Lab – Version 1 (Aescu HRL-1). The second purpose was to verify this prescription and compared its objective and subjective outcomes with a commonly used loudness equalization strategy: NAL-NL1. Speech intelligibility was tested using objective tests, while sound quality was evaluated by a subjective questionnaire.

## Methods

### Ethics Statement

The present study protocol was approved by Institutional Review Board on experimental ethics committee at National Taiwan University hospital (ID: 201003023R). All subjects provided written informed consent to participate.

### Rationale and design of the Mandarin-Based Fitting Strategy

**Figure 1:**
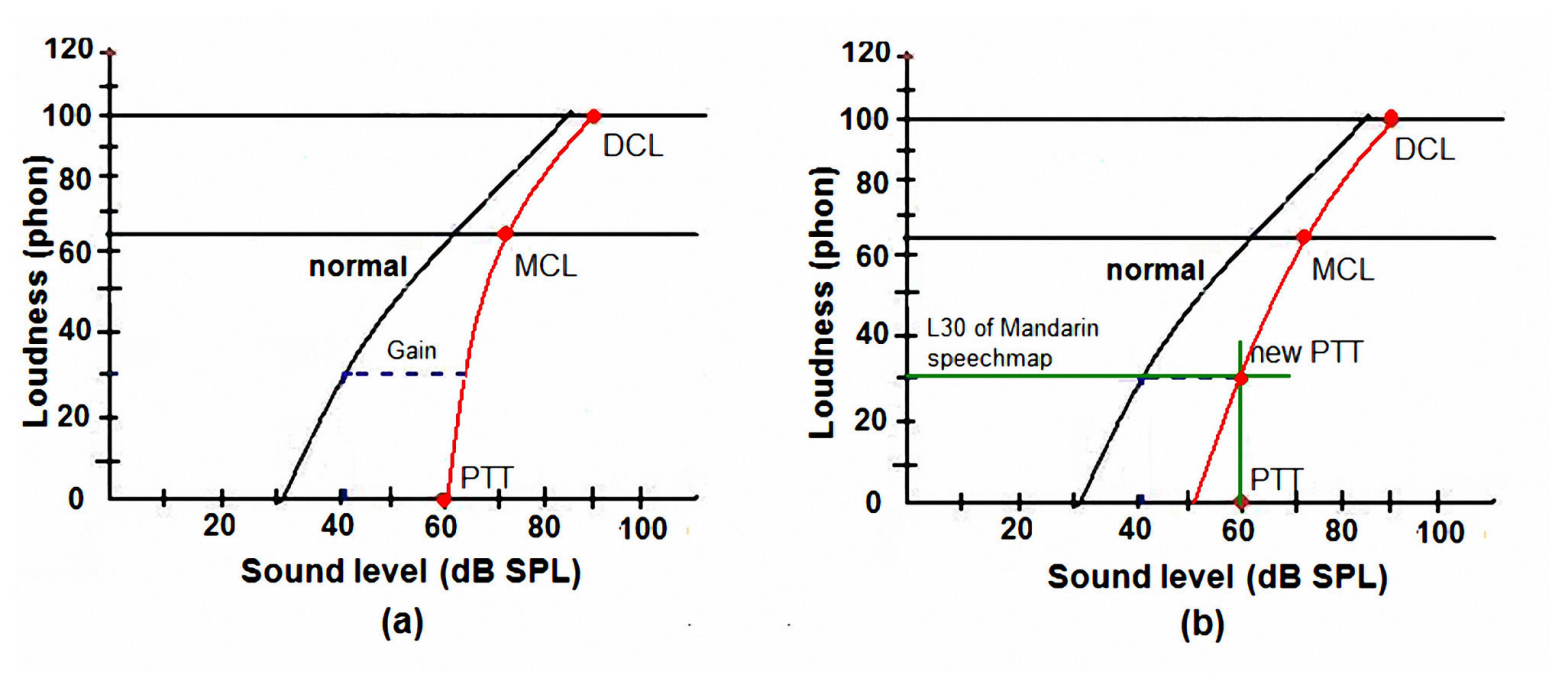
Example loudness-growth curves for Aescu HRL-1. (a). Original loudness-growth curve of a hearing-impaired individual. (b). Loudness-growth curve of a hearing-impaired individual based on the Mandarin speechmap.

The Aescu HRL-1 fitting strategy is a nonlinear amplification method developed on the rationale of loudness normalization, and it is threshold-based. The difference in the loudness-growth curves between NH and HI individuals is used to set the gain of the hearing aid. The loudness-growth curve of NH subjects was derived by applying spline nonlinear interpolation to the equal-loudness contours of Suzuki et al. [29,30]. Loudness-growth curves differ between HI and NH individuals. In this new fitting strategy, three points are used to derive the loudness-growth curve of a HI individual. These points are the sound pressure levels that produce loudness sensations of 0, 65, and 100 phon at various frequencies in HI individuals. The phon is the unit of measurement for loudness level: 0 phon usually corresponds to the hearing threshold level of pure-tone audibility, and is called the pure-tone threshold (PTT), 100 phon is assumed to be near the discomfort level (DCL), and 65 phon is near to the most comfortable level (MCL) for the HI individual in this prescription. The loudness-growth curve of the HI individual was then derived by using spline nonlinear interpolation to fit the curve through these three points. The compensation gain for hearing aids users is according the difference between NH and HI loudness-growth curve. Figure 1(a) provides an example of the obtained compensation gain: when the input sound level is 40 dB SPL, the gain is 25 dB based on the difference between these two growth curves. 

Previous studies showed that a fitting strategy based on loudness normalization may have some problems regarding the performance of speech intelligibility. In noisy environment, as both speech and background noise are dominated by energy in the low frequencies, the HI individuals could suffer from the effect of upward spread of masking, which compromises speech intelligibility [6]. Our preliminary results were similar in that loudness normalization provided excessive gain to low level sound input and over amplified background noise to cause discomfort [31]. This problem was improved in the design by modifying the prescription. Our strategy optimized the characteristics of Mandarin demonstrated in the speech map. The map is based on results obtained in 12 Taiwanese subjects (6 males and 6 females) speaking 300 phonemically and tonally balanced disyllabic words [26]. The L99 and L30 were defined as the top of the speech map as the SPL exceeded 1% of the time and the SPL exceeded 70% of the time [32]. According the concept of SII score, when the region of speech map above hearing threshold will result in SII score above 70 which results in a 100% score on the connected speech test (CST) [32]. Therefore, the L30 value of the Mandarin speech map [28] was used to modify this prescription to decrease the gain applied to low-level sound inputs. Figure 1(b) presents an example of how the loudness-growth curve was modified when the L30 value of the speech map is 30 phon at the indicated frequency. The PTT is mapped to 30 phon instead of 0 phone, and spline nonlinear interpolation is applied to the new PTT, MCL, and DCL to create the new loudness-growth curve of the HI individual. The compensatory gain in Aescu HRL-1 is the difference between the normal curve and this new loudness-growth curve of the HI individual. 

In addition, this modification decreases the gain for low input levels, and decreases the compression ratio when mapping to the WDRC architecture of hearing aids compared to the traditional methods of loudness normalization. van Buuren [33,34] showed that the sound quality was better for individuals with sensorineural hearing loss when the compression ratio was lower (i.e., closer to linear amplification). Therefore, in addition to providing good audibility for hearing-aid users, Aescu HRL-1 is expected to provide better sound quality.

### Subjects for verification

**Figure 2:**
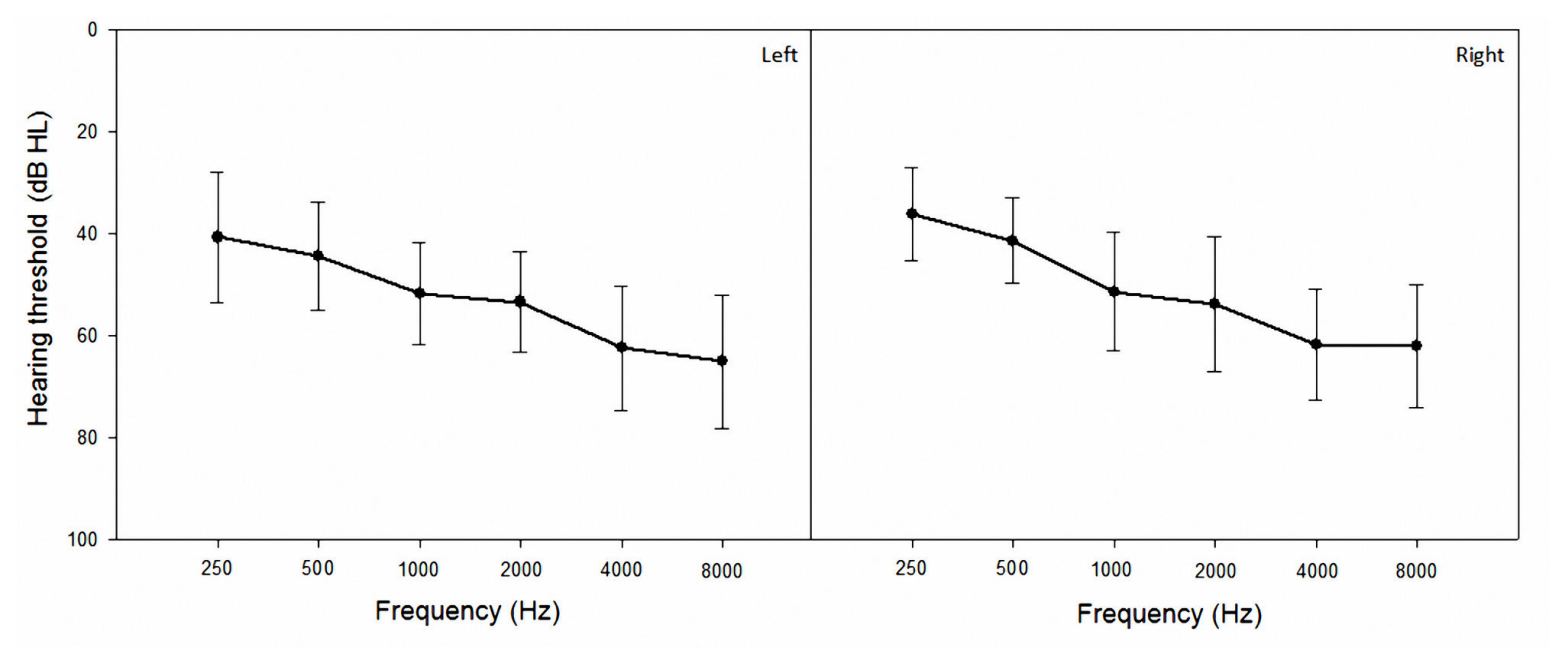
Hearing thresholds for the left and right ears of the 15 subjects. Data show mean and standard deviation values.

Fifteen subjects (eight male and seven females) whose native language is Mandarin participated in this study. The subjects aged from 24 to 66 years with a mean of 48 years. They all have postlingual, bilateral and symmetric sensorineural hearing loss. The average pure-tone hearing thresholds (500, 1000, 2000, and 4000 Hz) of both ears of each subject were all within the range 50–80 dB HL. Figure 2 shows the hearing thresholds for the left and right ears in these 15 subjects. Most of the subjects had a gradually sloping or flat hearing loss. None of the subjects had previous experience of hearing aids usage.

### Hearing-Aid Devices

The behind the ear (BTE) style hearing aids of FOCUS 4M, developed by Aescu in Taiwan, was used in this study. It is a 4-channel digital hearing aid and comprises eight filter bands and can provide independent compression in 4 channels. In addition, both the static and dynamic characteristics of the compression channels are adjustable, the bandwidth is from 200 to 5300 Hz, range of compression is from 1:1 to 1:5, and maximum output level is 129 dB SPL; additional features of FOCUS 4M includes directional microphone, noise reduction, feedback cancellation etc. FOCUS 4M can be set to the NAL-NL1 or Aescu-HRL1 prescriptions. 

### Subjective and objective outcome assessment

The NAL-NL1 and Aescu HRL-1 fitting strategies were compared using different objective and subjective methods. The speech recognition abilities in quiet and noise were measured with the Mandarin Monosyllable Recognition Test (MMRT) [35] and the Mandarin Hearing in Noise Test (MHINT) [36]. In MMRT, a set of 25-item word lists that exhibit familiarity, homogeneity, and phonemic balance was used, and each fitting strategy was evaluated for each subject by presenting speech level at 65 dB SPL. MHINT was also used to evaluate the benefits between NAL-NL1 and Aescu HRL-1, the first sentence was presented 10 dBA below the attenuator setting necessary for the noise to be presented at 65 dBA and first sentence was repeated. 

 The self-reported hearing instrument benefit and satisfaction measures were assessed using a self-designed sound-quality questionnaire (appendix S1) and the International Outcome Inventory for Hearing Aids (IOI-HA) [37]. The subject was asked to rate the sound quality and the scores on the IOI-HA questionnaires (appendix S2) for each fitting strategy on a scale from 4 (strongly agree) to 0 (strongly disagree), with 2 considered to be acceptable. After completing all items in the questionnaire, all subjects were also requested to provide a brief description for the sound quality of each fitting strategy.

### Experimental Design

The study was conducted with single-blind design. MMRT and MHINT were administered to each subject to assess the unaided speech recognition ability before hearing aid fitting. Then, bilateral hearing aids were both fitted alternatively with NAL-NL1 and Aescu HRL1, the order was counterbalanced. After fitting with one prescription, the subjects had 3–4 weeks to acclimatize to the amplified sounds of the hearing aids provided by each fitting strategy. Then the subjects’ aided speech recognition ability was assessed again and they were asked to complete the sound quality and IOI-HA questionnaires. After the tests, the subjects were fitted using another fitting strategy and the same experimental procedures as for the first fitting were repeated.

The Verifit hearing aid analyzer of Audioscan was used to evaluate whether the fitting is suitable or not based on speechmap. Speechmap creates a map of the amplified speech region within the residual auditory area using several simulated speech signals. Scollie and Seewald [38] provided evidence that the speechmap produced by simulated speech signals is a good predictor of the real speech output from compression-based hearing aids. For details of the test signals and analytical methods, the reader can refer to “Verifit User’s Guide Version 3.6” [39]. In hearing-aid fitting of this study, measurements of the real-ear insertion gain with the Audioscan Verifit based on speech presented at 50 dB SPL (soft), 65 dB SPL (moderate), and 75 dB SPL (loud) were used to verify the fitted frequency response characteristics related to the prescribed target. At a speech level of 65 dB SPL the LTASS and target insertion gain at each frequency differed by less than 3 dB, while at speech levels of 50 and 75 dB SPL the differences were less than 10 dB [10]. In order to ensure that the gain in each channel could be matched to a target setting, the functions of adaptive noise reduction, directional microphone, and volume control were turned off. These subjects were considered the experienced hearing aid users, therefore the target gain provided by fitting strategies were not reduced accordingly. The purpose of this setting ensured that the target gain of each prescription could be achieved. 

## Results

### Prescribed and Achieved Hearing-Aid Gain and Output

**Figure 3:**
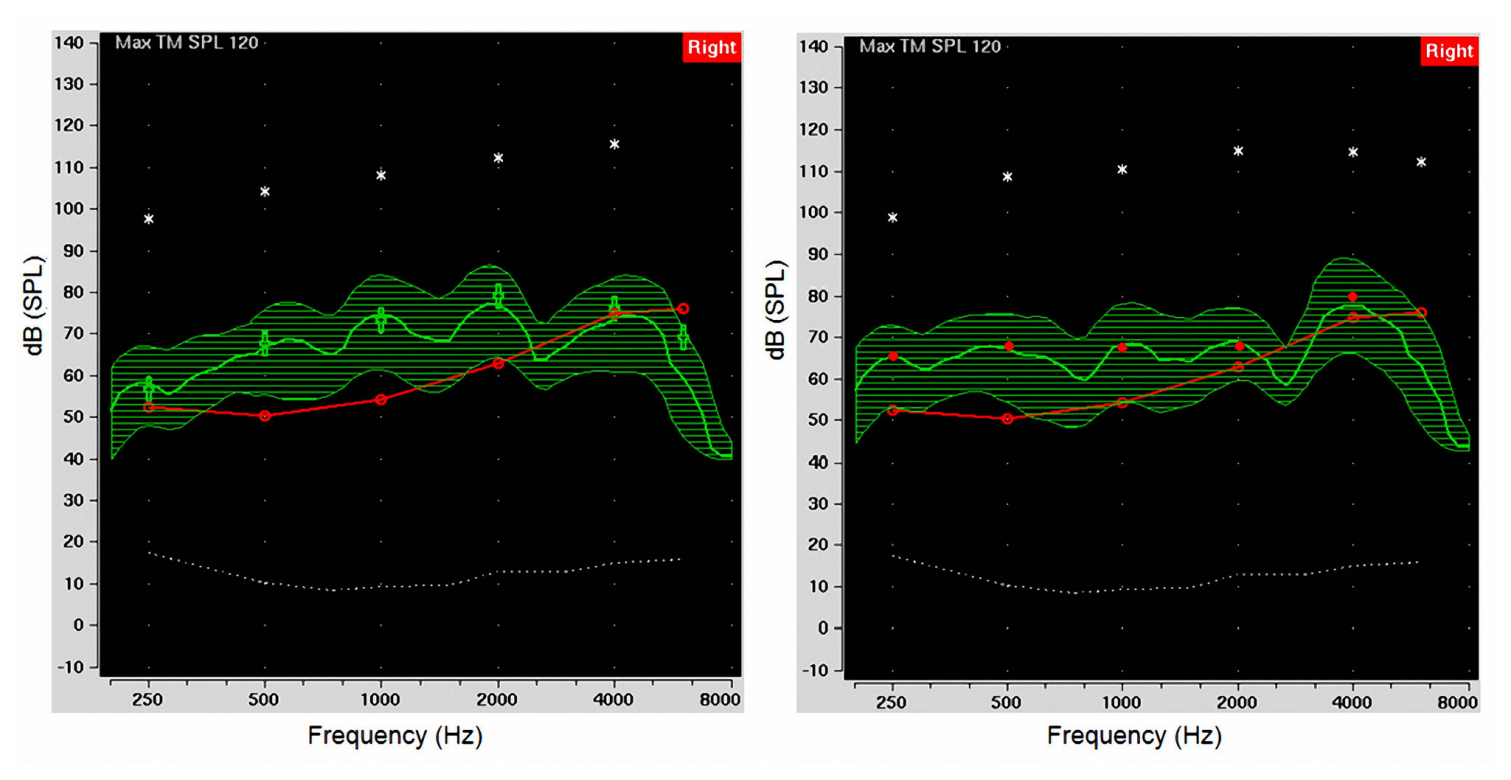
Example fitting results for the NAL-NL1 (left) and Aescu HRL-1 (right) prescriptions at a moderate speech level. The top, bottom, and middle curves of the green region represent L99, L30, and the LTASS in the speech map, respectively. The red curve is the hearing threshold and the white star symbols indicate the predicted discomfort levels.

**Figure 4:**
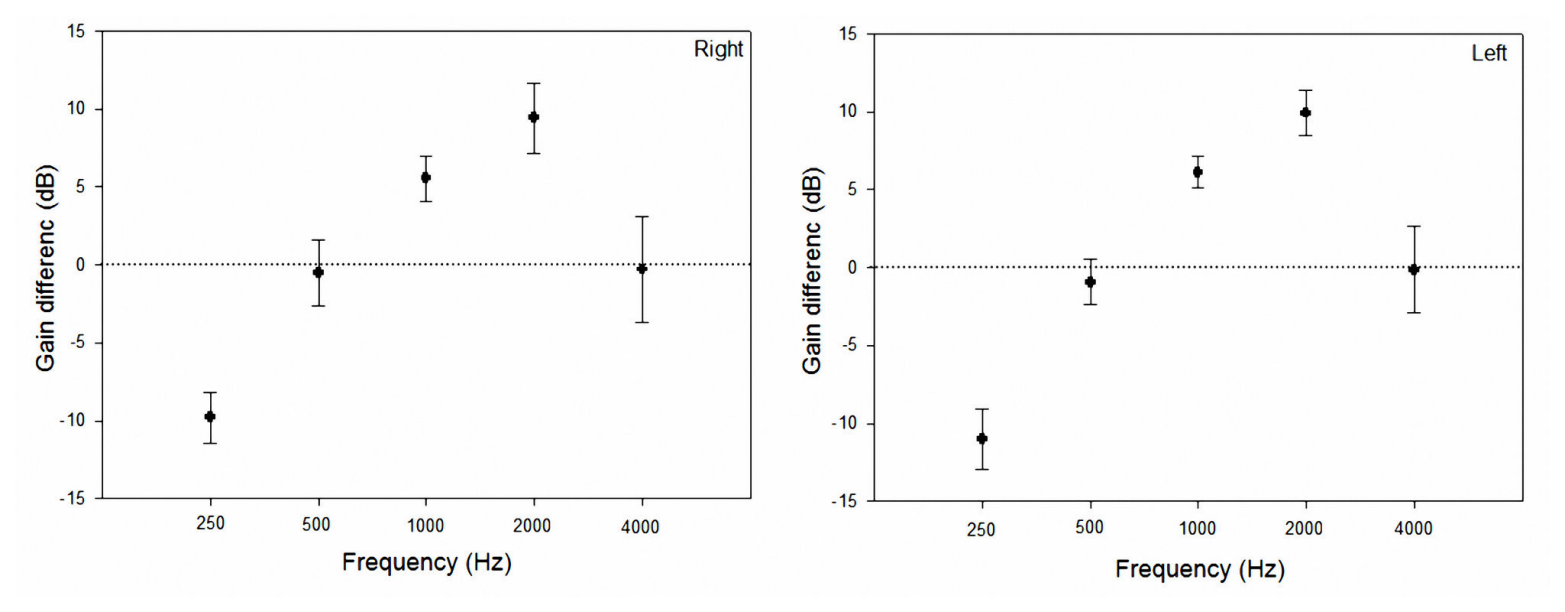
Differences in real-ear gain prescribed by NAL-NL1 and Aescu HRL-1 (NAL-NL1 – Aescu HRL-1) for a four-channel device for one input level in the right and left ears. The data are based on 30 ears. Data are mean and standard deviation values.

When the LTASS curve of speechmap fits the targets of the fitting strategy at each frequency, it means a good fitting based on the speechmap method. Figure 3 provides an example of fitting result for the NAL-NL1 (left) and Aescu HRL-1 (right) prescriptions at a moderate speech level. The top, bottom, and middle curves of the green region represent L99, L30, and the LTASS in the speechmap, respectively. The red curve is the hearing threshold and the white star symbols indicate the predicted discomfort levels. In addition, the green cross symbols (left) and red points (right) indicated the targets of NAL-NL1 and Aescu HRL-1 in Figure 3. The Figure showed that the amplified speechmap fits both the targets of NAL-NL1 and Aescu HRL-1. Figure 4 shows the differences in gains prescribed by NAL-NL1 and Aescu HRL-1 for the four-channel hearing aid at an input level of 65 dB SPL. Across all 15 subjects, Aescu HRL-1 prescribed higher gains at 250 and 500 Hz, while NAL-NL1 prescribed higher gains at 1000 and 2000 Hz. 

### Objective Assessment

#### MMRT

**Figure 5:**
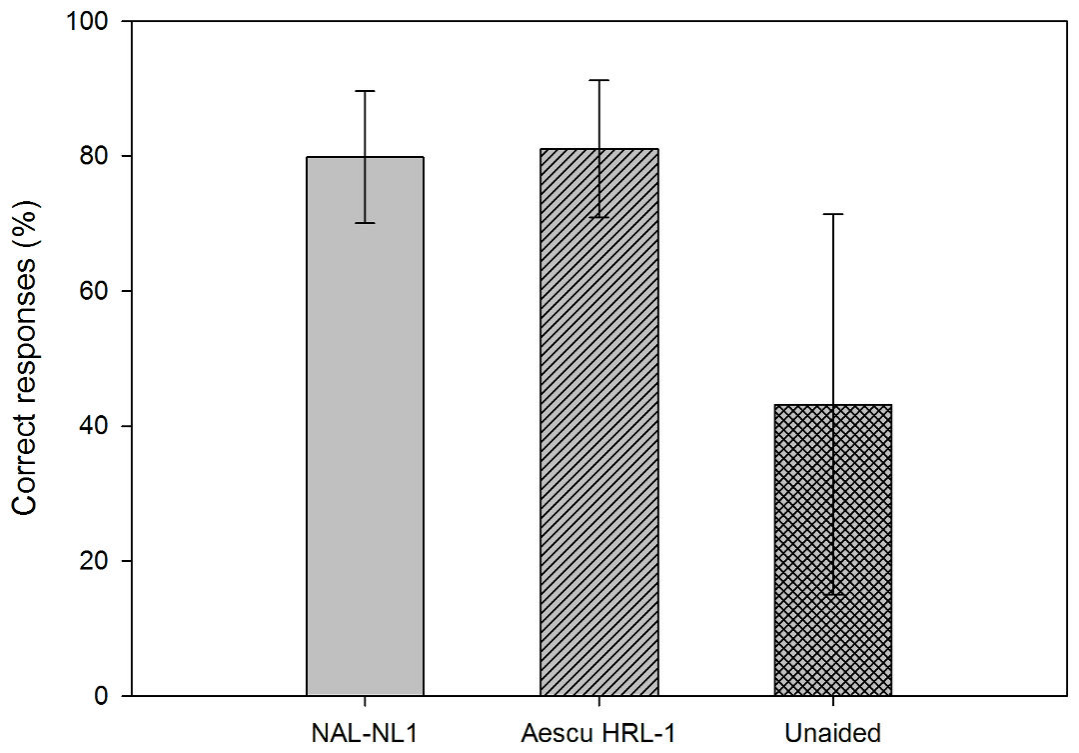
Percentages of correct MMRT responses among the 15 subjects at a moderate speech level. Data show mean and standard deviation values.

The correct rates for the speech recognition ability from the MMRT are shown in Figure 5. The mean correct rates for NAL-NL1, Aescu HRL-1, and unaided condition were 79.9%, 81.1%, and 43.2% respectively; the corresponding standard deviations were 9.8%, 10.2%, and 28.2%. The following probability values were obtained in paired-samples *t* tests for comparisons: unaided versus NAL-NL1, *p* < 0.01; unaided versus Aescu HRL-1, *p* < 0.01; and NAL-NL1 versus Aescu HRL-1, *p*=0.601. There was no statistical significance between these two fitting strategies despite scores improving significantly after amplification.

#### MHINT

**Figure 6:**
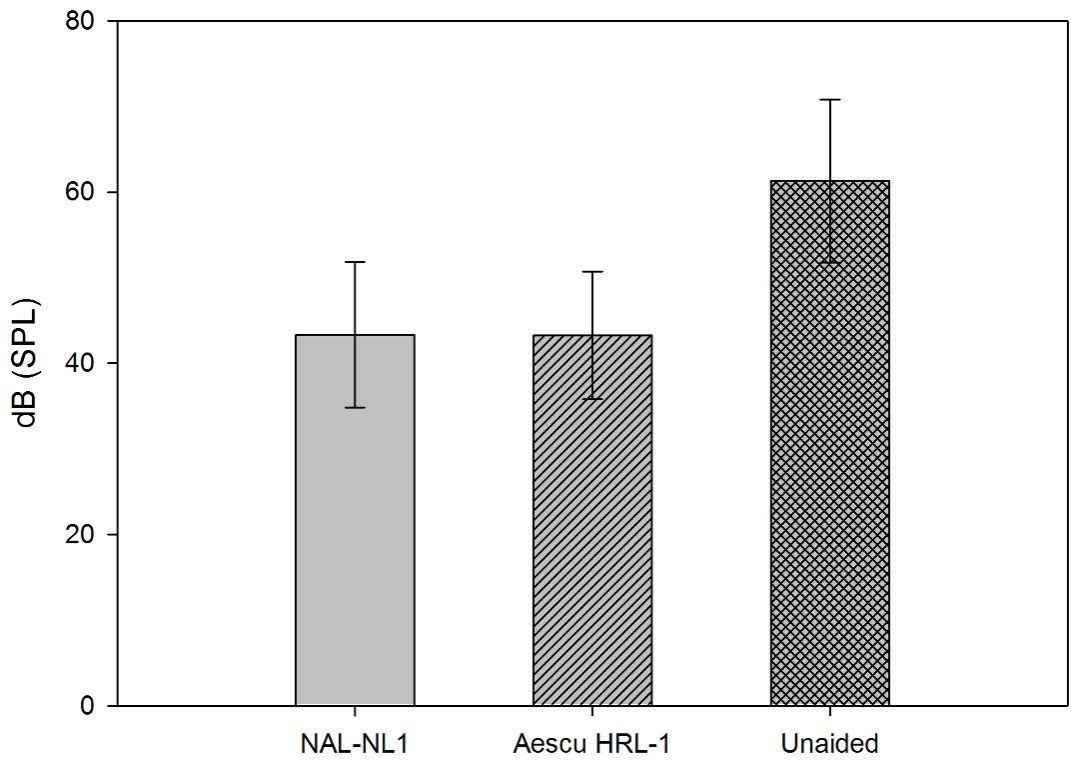
Presentation level required for a 50% correct rate in the MHINT in quiet for the 15 subjects at a moderate speech level. Data show mean and standard deviation values.

The mean presentation level required for a 50% correct score in the MHINT in quiet for the 15 subjects (see Figure 6) was 43.33 dB SPL for NAL-NL1, 43.23 dB SPL for Aescu HRL-1, and 61.30 dB SPL for unaided. The corresponding standard deviations were 8.49, 7.44, and 9.54 dB. The following probability values were obtained in paired-samples *t* tests for comparisons: unaided versus NAL-NL1, *p*<0.01; unaided versus Aescu HRL-1, *p*<0.01; and NAL-NL1 versus Aescu HRL-1, *p*=0.936. Again, no significant difference was found between these two strategies.

**Figure 7:**
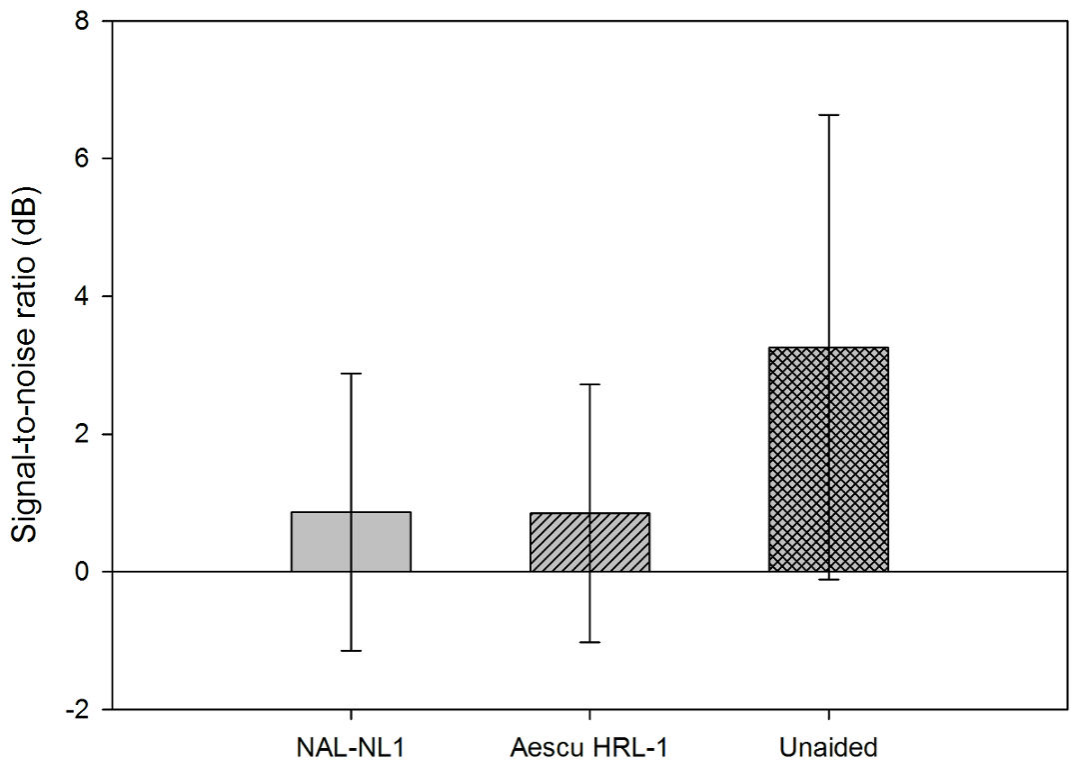
Signal-to-noise ratio required for a 50% correct rate in the MHINT in noise for the 15 subjects at a moderate speech level. Data show mean and standard deviation values.

The signal-to-noise ratios required for a 50% correct rate in the MHINT in noise for the 15 subjects (see Figure 7) were 0.87 dB for NAL-NL1, 0.85 dB for Aescu HRL-1, and 3.26 dB for unaided. The corresponding standard deviations were 2.01, 1.87, and 3.37 dB. The following probability values were obtained in paired-samples *t* tests for comparisons: unaided versus NAL-NL1, *p*=0.008; unaided versus Aescu HRL-1, *p*=0.003; and NAL-NL1 versus Aescu HRL-1, *p*=0.968. 

### Subjective Assessment

#### Sound quality

**Figure 8:**
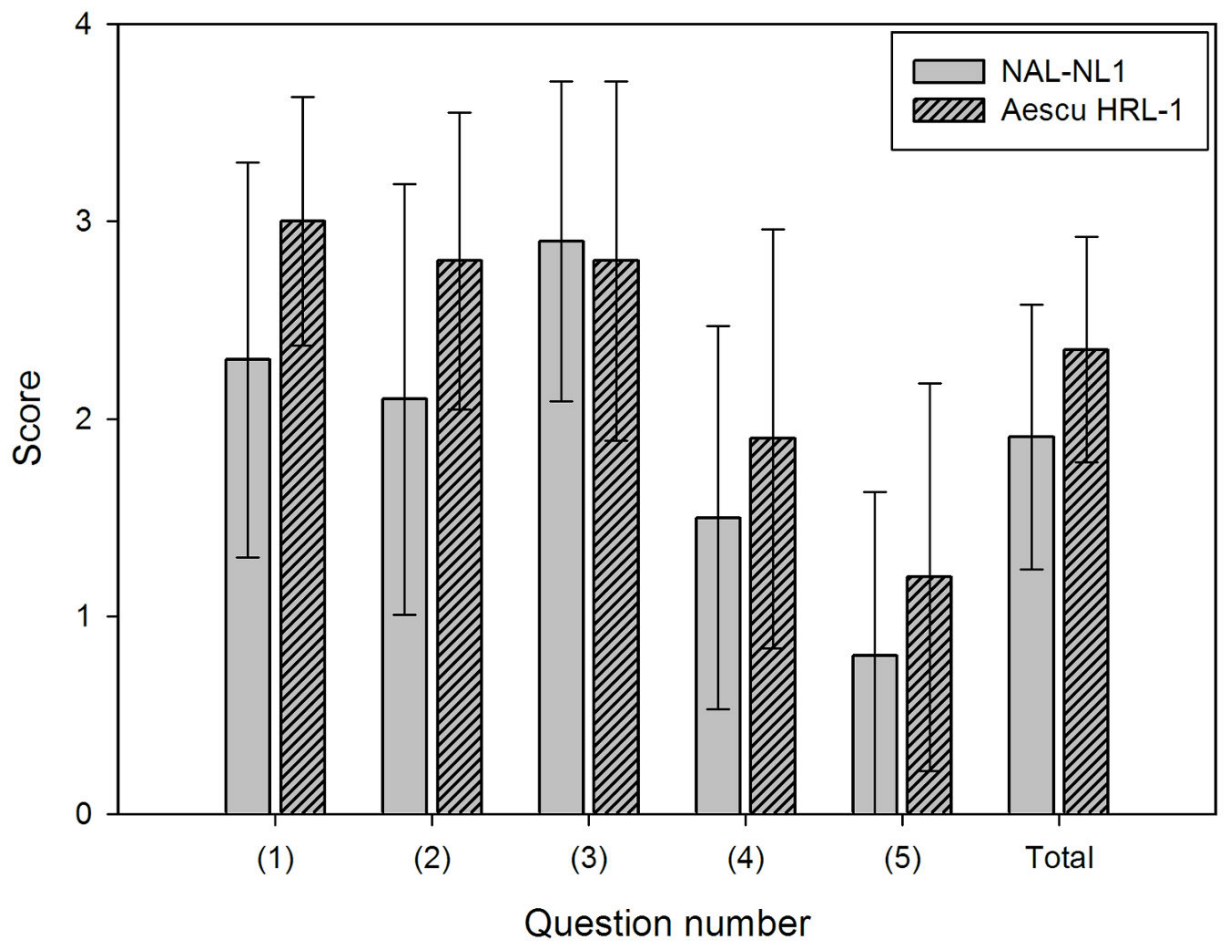
Scores on the sound-quality questionnaire for the 15 subjects. Data show mean and standard deviation values.

Figure 8 shows the scores on the sound-quality questionnaire for the two fitting strategies. A higher score indicates that the prescription was preferred. The scores for question 1 were 2.35±0.57 (mean±standard deviation) and 3.0±0.63 for NAL-NL1 and Aescu HRL-1, respectively; the corresponding scores for questions 2, 3, 4, and 5 were 2.1±1.09 and 2.80±0.75, 2.9±0.81 and 2.8±0.91, 1.50±0.97 and 1.9±1.06, 0.80±0.83 and 1.2±0.98, respectively. The mean score across the five questions was 1.91±0.67 for NAL-NL1 and 2.35±0.57 for Aescu HRL-1. The Wilcoxon non parametric test of NAL-NL1 versus Aescu HRL-1 showed a significant difference (p=0.027).

#### IOI-HA

**Figure 9:**
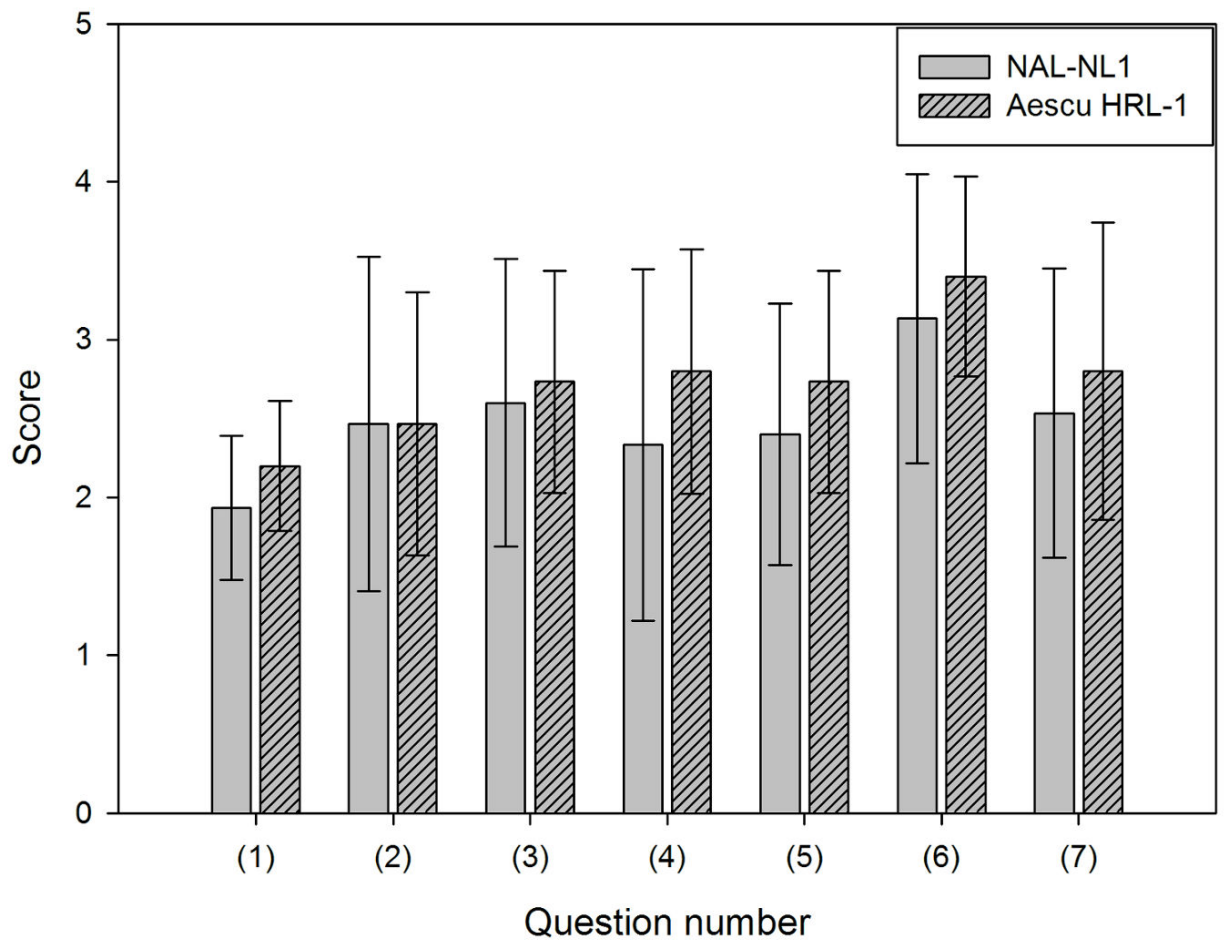
Scores on the IOI-HA questionnaire for the 15 subjects. Data are mean and standard deviation values. Questions 1 to 7 are listed in Appendix S2.

Figure 9 shows the scores for seven questions of the IOI-HA questionnaire. A higher score indicates that the prescription was more beneficial. The scores for question 1 were 1.93±0.46 and 2.20±0.41 for NAL-NL1 and Aescu HRL-1, respectively; the corresponding scores for questions 2, 3, 4, 5, 6, and 7 were 2.47±1.06 and 2.47±0.83, 2.60±0.91 and 2.73±0.70, 2.33±1.11 and 2.80±0.77, 2.40±0.83 and 2.73±0.70, 3.13±0.92 and 3.40±0.63, and 2.53±0.92 and 2.80±0.94, respectively. The average scores of these questions were showed a little higher for Aescu HRL-1, but used the Wilcoxon nonparametric test to compare these 7 questions between NAL-NL1 and Aesch HRL-1, the results showed no statistically significant difference (*p* > 0.05).

## Discussion

This study compared a modified loudness normalization prescription (Aescu HRL-1) with a loudness equalization prescription (NAL-NL1) implemented in a four-channel device, and applied to 15 subjects with flat and high-frequency hearing losses. The two prescriptions employed different amplification targets for these subjects. For example, for a moderate input level, the average gains prescribed by Aescu HRL-1 were 9.8, 0.5, and 0.3 dB higher at 250, 500, and 4000 Hz than those prescribed by NAL-NL1 across all 15 subjects. The trends of the target gains readily clarify the differences between Aescu HRL-1 and NAL-NL1, with the former based on the concept of loudness normalization amplification. 

The objective assessments revealed that the word intelligibility was significantly better for NAL-NL1 and Aescu HRL-1 than for the unaided condition, which means both fitting strategies improved the audibility of Mandarin words for HI individuals. However, the mean scores in the MMRT and MHINT did not differ significantly between the Aescu HRL-1 and NAL-NL1 fitting strategies, in other words, the Aescu HRL-1 can provide the same speech intelligibility for subjects NAL-NL1. Previous studies showed that the speech intelligibility was better for a loudness-equalization-based amplifier than for loudness normalization [5,6]. However, although Aescu HRL-1 is a loudness normalization based strategy with modification according to mandarin acoustic features, it actually produces similar performance to the “loudness equalization” group (NAL-NL1) in speech intelligibility. 

In addition to improve speech intelligibility, 88.4% of hearing-aid users seek improved sound quality [33]. Hence, improving sound quality is one of the important issues for fitting strategies in hearing-aid designs. Question 1 in the subjective questionnaire indicated that the perceived sound quality was better for Aescu HRL-1 than for NAL-NL1. This could be due to the use of a Mandarin speechmap to modify this prescription, which would decrease the compression ratio when Aescu HRL-1 is mapped to a WDRC amplifier, because a lower compression ratio (i.e., closer to linear amplification) can provide hearing-aid users with a better sound quality [33]. The responses to question 2 indicated that the sound was more natural for Aescu HRL-1 than for NAL-NL1, which could be due to the former being a normalization-based prescription, so the output sound is more similar to the sounds that the subjects heard previously. Question 3 indicated that the speech intelligibility was slightly clearer for NAL-NL1 than for Aescu HRL-1. However, the MMRT and MHINT revealed no significant differences in the word recognition scores. Therefore, overall these two fitting strategies provide subjects with similar speech intelligibilities. Questions 4 and 5 indicated that Aescu HRL-1 was more beneficial than NAL-NL1, but the average scores were lower than those for questions 1 to 3. This could have been due to the noise reduction, directional microphone, and volume control functions all being turned off, so the subjects could only use the amplifier function of the hearing aids in these two fitting strategies. However, the relative benefits of the two strategies might differ in the speech-in-noise condition. The mean scores of these five questions have showed that the Aescu HRL-1 provided significantly higher satisfaction than NAL-NL1 and have significant differences. In summary, the subjects found that Aescu HRL-1 provided a more natural, richer, and better sound quality than did NAL-NL1, and provided sounds that were more similar to those that they heard before experiencing hearing loss. 

Hearing loss will affect the performance of children when learning to speak [40,41]. When the processed speech (ie after amplification by hearing aids) causes too many distortions, it could affect speech learning, especially in preschool children. The Aescu HRL-1 strategy considers the difference in loudness growth curve between normal and impaired hearing to restoring loudness to normal. Therefore it would be expected to be more appropriate for preschool children.

In summary, this study has demonstrated that the amplification characteristics of the two prescriptions rely on marked differences in overall gains, with the subjects showing only minor preferences to either prescription. However, if the resulting amplification characteristics from the two prescriptions differ substantially, then the subjects showed a predominant preference for Aescu HRL-1, whose prescription aims at provide a natural sound of high quality. 

A limitation of this study is that the experiments were conducted with a moderate speech level only, without the use of the other speech levels to evaluate the performance in objective measurement test, and did not consider the interactions between fitting strategy and the other hearing aid functions, such as speech enhancement, feedback cancellation, and directional microphone. Therefore, the overall effectiveness of the Aescu HRL-1 combined with these algorithms needs to be investigated in future studies.

## Conclusion

The results show that the speech intelligibility of the Aescu HRL-1 was as good as NAL-NL1 in objective tests. In subjective evaluations the subjects generally preferred Aescu HRL-1, responding that this prescription provided richer, smoother, and more natural sound compared to NAL-NL1. This pilot study shows the potential of this new hearing-aid fitting prescription in providing a sound quality similar to that of a prescription based on loudness normalization, and speech intelligibility similar to that of a prescription based on loudness equalization.

## Supporting Information

Appendix S1 **The sound quality questionnaire.**
(PDF)

Appendix S2 **The international outcome inventory for hearing aids (IOI-HA) questionnaire.**
(PDF)

## References

[1] Corliss E, Burnett E, Kobal M, Bassin M (1968 ) The relative importance of frequency distortion and changes in time constants in the intelligibility of speech. IEEE Transactions on Audio and Electroacoustics 16: 36-39. doi:10.1109/TAU.1968.1161947.

[2] Preves D, Newton J (1989) The headroom problem and hearing aid performance. Hearing Journal 42: 19-26.

[3] Hawkins DB, Naidoo SV (1993) Comparison of sound quality and clarity with asymmetrical peak clipping and output limiting compression. J Am Acad Audiol 4: 221-228. PubMed: 8369539. 8369539

[4] Dillon H (1996) Tutorial compression? Yes, but for low or high frequencies, for low or high intensities, and with what response times? Ear Hear 17: 287–307. doi:10.1097/00003446-199608000-00001. PubMed: 8862967. 8862967

[5] Keidser G, Grant F (2003) Loudness normalization or speech intelligibility maximization? Differences in clinical goals, issues, and preferences. Hearing Review 10: 14-25.

[6] Keidser G, Grant F (2001) Comparing loudness normalization (IHAFF) with speech intelligibility maximization (NAL-NL1) when implemented in a two-channel device. Ear Hear 22: 501-515. doi:10.1097/00003446-200112000-00006. PubMed: 11770672. 11770672

[7] Valente M, Hosford-Dunn H, Roeser RJ (2007) Audiology treatment: Thieme Medical Pub.

[8] Cox RM (1995) Using loudness data for hearing aid selection: The IHAFF approach. Hearing Journal 48: 10. doi:10.1097/00025572-199504000-00001.

[9] Seewald R, Ramji K, Sinclair S, Moodie K, Jamieson D (1993) Computer-assisted implementation of the desired sensation level method for electroacoustic selection and fitting in children: version 3.1 user’s manual. Hearing Health Care Research Unit Technical Report 2

[10] Dillon H (2001) Hearing aids. Thieme Medical, New York.

[11] Keidser G, Dillon H, Flax M, Ching T, Brewer S (2011) The NAL-NL2 prescription procedure. Audiology Research 1: e24. 26557309 10.4081/audiores.2011.e24 PMC4627149

[12] Moore B, Glasberg B (1997) A model of loudness perception applied to cochlear hearing loss. Auditory Neuroscience 3: 289-311.

[13] Byrne D, Dillon H, Ching T, Katsch R, Keidser G (2001) NAL-NL1 procedure for fitting nonlinear hearing aids: characteristics and comparisons with other procedures. J Am Acad Audiol 12: 37–51. PubMed: 11214977. 11214977

[14] Ching TYC, Dillon H, Byrne D (1998) Speech recognition of hearing-impaired listeners: Predictions from audibility and the limited role of high-frequency amplification. J Acoust Soc Am 103: 1128–1140. doi:10.1121/1.421224. PubMed: 9479766. 9479766

[15] Bench R, Doyle J (1979) The BKB/A (Bamford-Kowal-Bench/Australian version) sentence lists for hearing-impaired children. Victoria: La Trobe University.

[16] Keidser G, Grant F (1999) Evaluation of loudness equalisation versus loudness normalisation. Hearing Aid Amplification for the New Millenium Sydney.

[17] Valente M (2002) Strategies for selecting and verifying hearing aid fittings: Thieme Medical Pub.

[18] Moore BC, Glasberg BR, Stone MA (2010) Development of a new method for deriving initial fittings for hearing aids with multi-channel compression: CAMEQ2-HF. Int J Audiol 49: 216-227. doi:10.3109/14992020903296746. PubMed: 20151930. 20151930

[19] Moore BC, Füllgrabe C (2010) Evaluation of the CAMEQ2-HF Method for Fitting Hearing Aids With Multichannel Amplitude Compression. Ear Hear 31: 657-666. PubMed: 20526199. 20526199 10.1097/AUD.0b013e3181e1cd0d

[20] Moore BC, Füllgrabe C, Stone MA (2010) Effect of spatial separation, extended bandwidth, and compression speed on intelligibility in a competing-speech task. J Acoust Soc Am 128: 360–371. doi:10.1121/1.3436533. PubMed: 20649230. 20649230

[21] Moore BC, Sek A (2013) Comparison of the CAM2 and NAL-NL2 Hearing Aid Fitting. Methods - Ear and Hearing 34: 83-95. doi:10.1097/AUD.0b013e3182650adf. 22878351

[22] Wikipedia (2011 ) List of languages by number of native speakers. Wikipedia.

[23] Byrne D, Dillon H, Tran K, Arlinger S, Wilbraham K et al. (1994) An international comparison of long-term average speech spectra. Journal of the Acoustical Society of America 96: 2108-2120. doi:10.1121/1.410152.

[24] McCullough JA, Tu C, Lew HL (1993) Speech-spectrum analysis of Mandarin: implications for hearing-aid fittings in a multi-ethnic society. J Am Acad Audiol 4: 50-52. PubMed: 8422484. 8422484

[25] Barbara Lust JG (1999) Lexical Anaphors and Pronouns in Selected South Asian Languages. Walter de Gruyter.

[26] Jing Chen TSQ, Wu Xihong H, Huang Qiang, Huang Ying, Liang Li et al. (2008) Frequency importance function of Mandarin Chinese speech. Journal of the Acoustical Society of America 123: 3323. doi:10.1121/1.2933804.

[27] Yeh Shiu-Huei. S-TY (6, 2005) Analysis of frequency importance function for Mandarin Speech perception, Master dissertation. National Yang-Ming University.

[28] Lee J (7, 2008) Adjust loudness model compensation strategy according to Mandarin speech map, Master dissertation, National Yang-Ming University.

[29] Suzuki Y, Takeshima H (2004) Equal-loudness-level contours for pure tones. J Acoust Soc Am 116: 918-933. doi:10.1121/1.1763601. PubMed: 15376658. 15376658

[30] Takeshima H, Suzuki Y, Ozawa K, Kumagai M, Sone T (2003) Comparison of loudness functions suitable for drawing equal-loudness-level contours. Acoustical Science and Technology 24: 61-68. doi:10.1250/ast.24.61.

[31] Jhou Y-M (2007) Development and achievement of hearing aid compensation strategy with loudness model, Master dissertationNational Yang-Ming University.

[32] Cole B (2005) A Brief History of "Speechmapping" at Audioscan. Dorchester, Ontario.

[33] van Buuren RA, Festen JM, Houtgast T (1999) Compression and expansion of the temporal envelope: Evaluation of speech intelligibility and sound quality. J Acoust Soc Am 105: 2903–2913. doi:10.1121/1.426943. PubMed: 10335639. 10335639

[34] Souza PE (2002) Effects of compression on speech acoustics, intelligibility, and sound quality. Trends in Amplification 6: 131-165. doi:10.1177/108471380200600402. 25425919 PMC4168964

[35] Tsai KS, Tseng LH, Wu CJ, Young ST (2009) Development of a mandarin monosyllable recognition test. Ear Hear 30: 90-99. doi:10.1097/AUD.0b013e31818f28a6. PubMed: 19125031. 19125031

[36] Wong LLN, Soli SD, Liu S, Han N, Huang MW (2007) Development of the mandarin hearing in noise test (MHINT). Ear Hear 28: 70S–74S. doi:10.1097/AUD.0b013e31803154d0. PubMed: 17496652. 17496652

[37] Cox RM, Alexander GC (2002) The International Outcome Inventory for Hearing Aids (IOI-HA): psychometric properties of the English version: El Inventario International de Resultados para Auxiliares Auditivos (IOI-HA): propiedades psicometricas de la version en ingles. Int J Audiol 41: 30-35. doi:10.3109/14992020209101309. PubMed: 12467367. 12467367

[38] Scollie SD, Seewald RC (2002) Evaluation of electroacoustic test signals I: Comparison with amplified speech. Ear Hear 23: 477-487. doi:10.1097/00003446-200210000-00009. PubMed: 12411780. 12411780

[39] Audioscan (2010) Verifit®User's Guide version 3.6 audioscan ®.

[40] Geers A, Moog J (1989) Factors predictive of the development of literacy in profoundly hearing-impaired adolescents. The Volta. ReView.

[41] Mogford K (1993) Oral language acquisition in the prelinguistically deaf. Language Development in Exceptional Circumstances: 110-131.

