# Supplementary material for: Development and Preliminary Verification of a Mandarin-Based Hearing-Aid Fitting Strategy
Source: PLoS One. 2013 Nov 20;8(11):e80831. doi: 10.1371/journal.pone.0080831 (PMC3835675; doi:10.1371/journal.pone.0080831)
Supplement: Appendix S1 — The sound quality questionnaire. (PDF) [file pone.0080831.s001.pdf]

Appendix S1

Sound-quality questionnaire

Name： ____________________ **Gender：** □ Male □ Female

Birthdate： __________________ **Date：** _________________

Please fill in this questionnaire according to your own feelings and personal preferences during the previous month. Thank you for your cooperation.

		**Strongly agree**	**Agree**	**Neither agree nor disagree**	**Disagree**	**Strongly disagree**
1	I think this hearing aid provides high sound quality.					
2	I think this hearing aid provides a natural sound.					
3	I can hear very clearly when I use this hearing aid.					
4	I feel comfortable when I use this hearing aid.					
5	I can’t hear noise when I use this hearing aid.					
