# Supplementary material for: Development and Preliminary Verification of a Mandarin-Based Hearing-Aid Fitting Strategy
Source: PLoS One. 2013 Nov 20;8(11):e80831. doi: 10.1371/journal.pone.0080831 (PMC3835675; doi:10.1371/journal.pone.0080831)
Supplement: Appendix S2 — The international outcome inventory for hearing aids (IOI-HA) questionnaire. (PDF) [file pone.0080831.s002.pdf]

Appendix S2

INTERNATIONAL OUTCOME INVENTORY – HEARING AIDS (IOI-HA)

1. Think about how much you used your present hearing aid(s) over the past two weeks. On an average day, how many hours did you use the hearing aid(s)?

none	less than 1 hours a day	1 to 4 hours a day	4 to 8 hours a day	more than 8 hours a day
❑	❑	❑	❑	❑

2. Think about the situation where you most wanted to hear better, before you got your present hearing aid(s). Over the past two weeks, how much has the hearing aid helped in that situation?

helped not at all	helped slightly	helped moderately	helped quite a lot	helped very much
❑	❑	❑	❑	❑

3. Think again about the situation where you most wanted to hear better. When you use your present hearing aid(s), how much difficulty do you STILL have in that situation?

very much difficulty	quite a lot of difficulty	moderate difficulty	slight difficulty	no difficulty
❑	❑	❑	❑	❑

4. Considering everything, do you think your present hearing aid(s) is worth the trouble?

not at all worth it	slightly worth it	moderately worth it	quite a lot worth it	very much worth it
❑	❑	❑	❑	❑

5. Over the past two weeks, with your present hearing aid(s), how much have your hearing difficulties affected the things you can do?

affected very much	affected quite a lot	affected moderately	affected slightly	affected not at all
❑	❑	❑	❑	❑

6. Over the past two weeks, with your present hearing aid(s), how much do you think other people were bothered by your hearing difficulties?

bothered very much	bothered quite a lot	bothered moderately	bothered slightly	bothered not at all
❑	❑	❑	❑	❑

7. Considering everything, how much has your present hearing aid(s) changed your enjoyment of life?

worse	no change	slightly better	quite a lot better	very much better
❑	❑	❑	❑	❑

English Version
